# Comparison of eight complete plastid genomes from three moss families Amblystegiaceae, Calliergonaceae and Pylaisiaceae

**DOI:** 10.1080/23802359.2020.1797548

**Published:** 2020-07-30

**Authors:** Wei Sheng, Xin-Rui Yue, Na Li, Yang Liu, Yu-Huan Wu

**Affiliations:** a Key Laboratory of Forest Ecology and Management, Institute of Applied Ecology, Chinese Academy of Sciences, Shenyang, China; b Fairy Lake Botanical Garden, Shenzhen and Chinese Academy of Sciences, Shenzhen, China; c Graduate School, University of Chinese Academy of Sciences, Beijing, China; d College of Life and Environmental Sciences, Hangzhou Normal University, Hangzhou, China

**Keywords:** Plastid genome, Amblystegiaceae, Calliergonaceae, phylogenetic analysis

## Abstract

We sequenced and assembled eight complete plastid genomes from three closely related pleurocarpous moss families: *Amblystegium serpens*, *Campyliadelphus stellatus*, *Cratoneuron filicinum*, *Drepanocladus aduncus*, and *Leptodictyum humile* (Amblystegiaceae), *Calliergon sarmentosum* and *Warnstorfia exannulata* (Calliergonaceae), and *Calliergonella cuspidata* (Pylaisiaceae). The newly generated plastid genomes range from 124,256 to 124,819 bp, with two inverted repeat regions (9,624–9,696 bp) separated by a large single-copy region (86,422–86,924 bp) and a small single-copy region (18,430–18,514 bp). All these plastid genomes encode 116 unique genes including 82 protein-coding genes, 30 tRNA genes and four rRNAgenes. The overall GC content is between 28.6%–29.3%. Phylogenetic analysis showed that all Amblystegiaceae species *Amblystegium serpens*, *Campyliadelphus stellatus*, *Cratoneuron filicinum*, *Drepanocladus aduncus*, *Leptodictyum humile*, and *Sanionia uncinata* clustered in one clade, which is sister to the Pylaisiaceae species *Calliergonella cuspidata*. The two Calliergonaceae species *Calliergon sarmentosum* and *Warnstorfia exannulata* form a clade and is sister to Amblystegiaceae and Pylaisiaceae.

The moss family Amblystegiaceae was first recognized in 1885 and was placed close to Hypnaceae (Kindberg 1885). Then the family was thoroughly redescribed by Roth (1899). Since the second edition of the book Musci in *Natürlichen Pflanzenfamilien* (Brotherus 1925), Amblystegiaceae was universally accepted as a family. Amblystegiaceae were traditionally circumscribed by their mostly single and long costa in leaf, cylindrical and curved spore capsule, and their preference for moist biotope (Hedenäs and Vanderpoorten 2007). Molecular phylogenetic studies based on chloroplast (*trnL*-*trnF* and *atpB*-*rbcL*) and nuclear (*ITS*) markers have provided strong evidence that the family should be splitted into Amblystegiaceae and Calliergonaceae (Hedenäs et al. 2005; Vanderpoorten et al. 2002a, 2002b, 2003), this treatment has been followed by the classification of the Bryophyta of Goffinet & Buck (2004), Goffinet et al. (2008), and Frey & Stech (2009). Considering the complicated relationship of Amblystegiaceae, we selected eight species (Table 1) belonging to eight genera of traditional Amblystegiaceae based on morphological classification (Vitt 1984), and assembled and annotated their complete plastid genomes.

**Table 1.** Voucher information of the eight samples.

Family	Species	Collection number	Locality	GenBank accession numbers
Amblystegiaceae	*Amblystegium serpens* (Hedw.) Schimp.	Wu 13071242	Inner Mongolia, China. (51°20′34″N, 120°51′59″E)	MT354751
	*Cratoneuron filicinum* (Hedw.) Spruce	Wu 2012072132	Jilin, China. (42°24′22″N, 128°08′29″E)	MT354755
	*Campyliadelphus stellatus* (Hedw.) Kanda	Wu 2012072249	Jilin, China. (42°24′22″N, 128°08′29″E)	MT354754
	*Drepanocladus aduncus* (Hedw.) Warnst.	Zuo 2012549	Yunnan, China. (27°32′08″N, 98°49′52″E)	MT354756
	*Leptodictyum humile* (P. Beauv.) Ochyra	Wu 120731009	Jilin, China. (42°24′22″N, 128°08′29″E)	MT354757
Calliergonaceae	*Calliergon sarmentosum* (Wahlenb.) Kindb.	Wu 2012072247	Jilin, China. (42°24′22″N, 128°08′29″E)	MT354753
	*Warnstorfia exannulata* (Schimp.) Loeske	Wu 2012072282	Jilin, China. (42°24′22″N, 128°08′29″E)	MT354758
Pylaisiaceae	*Calliergonella cuspidata* (Hedw.) Loeske	Zeng 2012092206	Heilongjiang, China. (52°56′29″N, 122°51′30″E)	MT354752

These voucher specimens have been deposited at the herbarium of Hangzhou Normal University (HTC). The classfication followed Goffinet et al. (2008) and Paulo et al. (2018).

The total DNA was extracted using the modified CTAB method (Forrest et al. 2011). Genome sequencing was performed using the BGISEQ platform (BGI, Shenzhen, China), and about 3 Gb raw sequence data were generated for each sample. The sequence reads were assembled using GetOrganelle (Jin et al. 2018), which relies on SPAdes (Bankevich et al. 2012), Bowtie2 (Langmead and Salzberg 2012), and BLAST+ (Camacho et al. 2009). For the annotation, the program PGA (Qu et al. 2019) was used, with the plastid genome of *Sanionia uncinata* (NC_025668.1; Park et al. 2018), an Amblystegiaceae specie as the reference.

The newly generated plastid genomes are between 124,256 to 124,819 bp in size, and with two inverted repeat regions (9,624–9,696 bp) separated by a large single-copy region (86,422–86,924 bp) and a small single-copy region (18,430–18,514 bp). The plastid genome each encodes 116 unique genes including 82 protein-coding genes, 30 tRNA genes, and four rRNA genes. Among these, five tRNA genes (*trn*A-UGC, *trn*I-GAU, *trn*N-GUU, *trn*R-ACG, and *trn*V-GAC) and four rRNA genes are duplicated in the IR regions. A total of 16 genes harbor intron, 14 genes (*atpF*, *ndhA*, *ndhB*, *rpoC1*, *rps12*, *rpl16*, *rpl2*, *ycf66*, *trn*A-UGC, *trn*G-UCC, *trn*I-GAU, *trn*K-UUU, *trn*L-UAA, and *trn*V-UAC) contain one intron, and two genes (*ycf3* and *clpP*) contain two introns. The overall GC content of these plastid genomes is between 28.6% to 29.3%.

To reconstruct the phylogenetic relationships of these eight species within the Hypnales, we composed a data matrix of plastid genes derived from the newly seuqenced plastid gneomes, the plastid genome of *Sanionia uncinata* (Park et al. 2018), and plastid protein-coding genes from 12 genera of Hypnales (Liu et al. 2019). A total of 82 plastid protein-coding genes were extracted, and aligned with MAFFT v7.355 (Nakamura et al. 2018). The Maximum Likelihood tree was calculated under the parameter of PROTGAMMAAUTO using RAxML v8.2.11 (Stamatakis 2014). The resulted phylogenetic tree reveals the Amblystegiaceae species *Amblystegium serpens*, *Campyliadelphus stellatus*, *Cratoneuron filicinum*, *Drepanocladus aduncus*, *Leptodictyum humile*, and *Sanionia uncinata* cluster together, and is sister to the Pylaisiaceae *Calliergonella cuspidata*, and the Calliergonaceae species *Calliergon sarmentosum* and *Warnstorfia exannulata* form a clade and is sister to Amblystegiaceae and Pylaisiaceae (Figure 1). This result is consisitent with former studies of Hedenäs et al. (2005), Hedenäs & Vanderpoorten (2007), and Vanderpoorten et al. (2002a, 2002b), supporting the traditional family Amblystegiaceae should be splitted into Amblystegiaceae and Calliergonaceae. 

**Figure 1.**
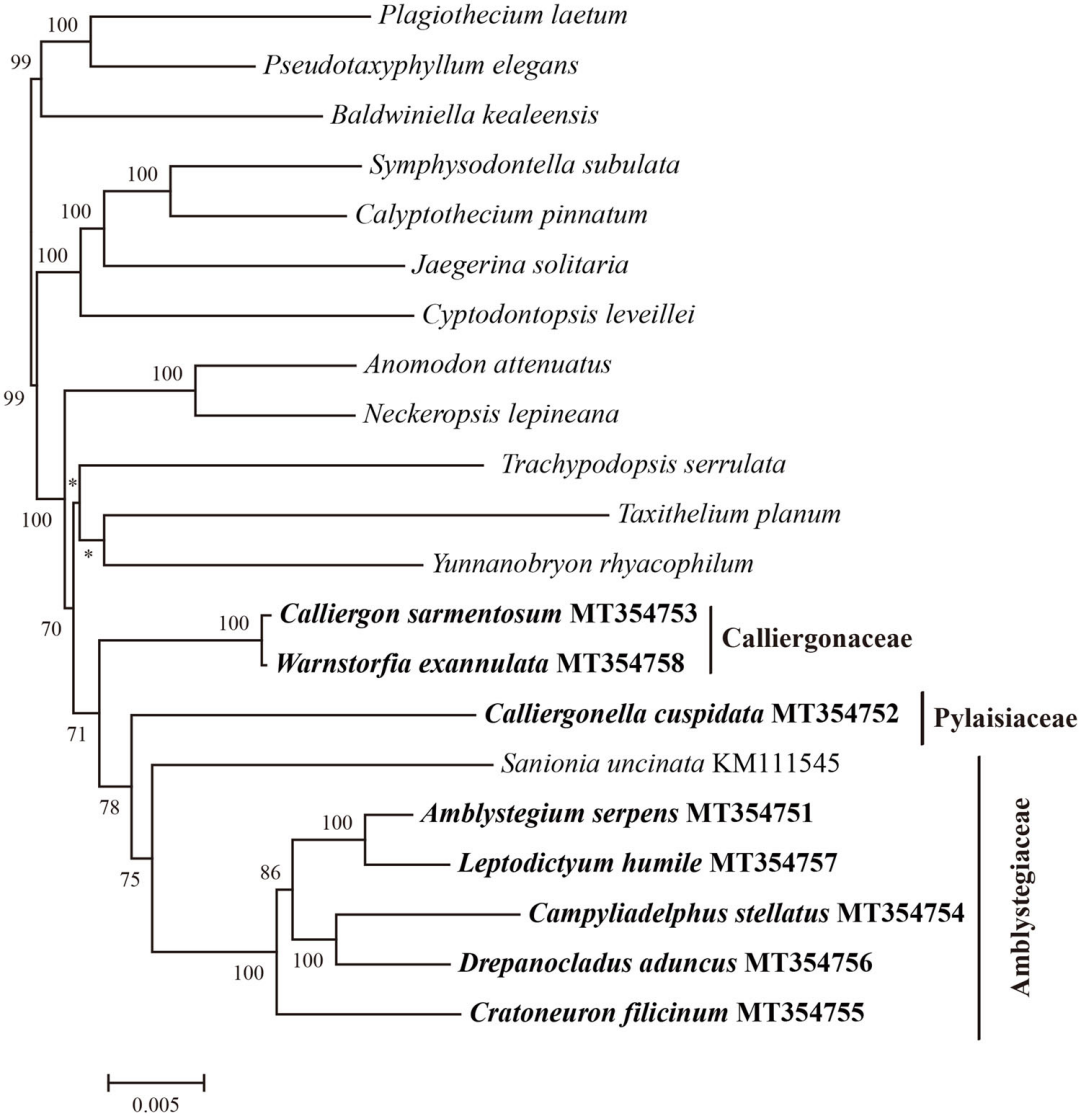
The maximum likelihood tree of 21 Hypnales species based on 82 plastid protein-coding genes. The numbers above the branches are bootstrap support values, * indicate bootstrap is < 50. The newly seqeunced eight species *Amblystegium serpens*, *Campyliadelphus stellatus*, *Cratoneuron filicinum*, *Drepanocladus aduncus*, *Leptodictyum humile*, *Calliergon sarmentosum*, *Warnstorfia exannulata*, and *Calliergonella cuspidata* are in bold. The data of the species without GenBank accession numbers were retrieved from the study of Liu et al. (2019). The classfication followed Goffinet et al. (2008) and Paulo et al. (2018).

## Data Availability

The annotated plastid genomes have been deposited in the GenBank database (accession number MT354751–MT354758). The raw genomic NGS read data for assembling the plastid genomes have been deposited in the NCBI Sequence Read Archive (SRA; accession no. SRP260466; https://www.ncbi.nlm.nih.gov/sra/?term=SRP260466).
